# Effects of TiN Top Electrode Texturing on Ferroelectricity in Hf_1–*x*_Zr_*x*_O_2_

**DOI:** 10.1021/acsami.1c01734

**Published:** 2021-02-24

**Authors:** Robin Athle, Anton E. O. Persson, Austin Irish, Heera Menon, Rainer Timm, Mattias Borg

**Affiliations:** † Electrical and Information Technology, Lund University, Box 118, 22 100 Lund, Sweden; ‡ Division of Synchrotron Radiation Research, Lund University, Box 118, 22 100 Lund, Sweden; § NanoLund Lund University, Box 118, 22 100 Lund, Sweden

**Keywords:** hafnium oxide, III−V, ferroelectric FET, ferroelectric tunnel junction, thin films, CMOS integration

## Abstract

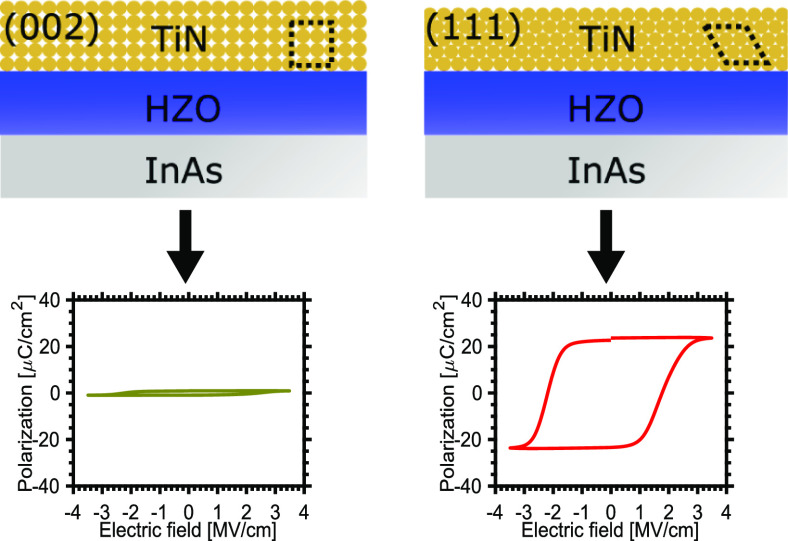

Ferroelectric memories based on hafnium oxide are an attractive alternative to conventional memory technologies due to their scalability and energy efficiency. However, there are still many open questions regarding the optimal material stack and processing conditions for reliable device performance. Here, we report on the impact of the sputtering process conditions of the commonly used TiN top electrode on the ferroelectric properties of Hf_1–*x*_Zr_*x*_O_2_. By manipulating the deposition pressure and chemistry, we control the preferential orientation of the TiN grains between (111) and (002). We observe that (111) textured TiN is superior to (002) texturing for achieving high remanent polarization (*P*_r_). Furthermore, we find that additional nitrogen supply during TiN deposition leads to >5× greater endurance, possibly by limiting the scavenging of oxygen from the Hf_1–*x*_Zr_*x*_O_2_ film. These results help explain the large *P*_r_ variation reported in the literature for Hf_1–*x*_Zr_*x*_O_2_/TiN and highlights the necessity of tuning the top electrode of the ferroelectric stack for successful device implementation.

## Introduction

Ferroelectricity in HfO_2_ has since its discovery in 2011^1^ been attracting strong interest for applications in nonvolatile memories and negative-capacitance transistors due to its strong remanent polarization (*P*_r_ ∼ 20–30 μC/cm^2^) and high coercive field (*E*_c_ ∼ 1–2 MV/cm^2^), as well as being compatible with and already used in complementary metal-oxide semiconductor technology. In addition, ferroelectric (FE) HfO_2_ can exhibit memristive behavior in ferroelectric tunnel junctions^2^ (FTJs) and ferroelectric field effect transistors (FeFETs),^3^ which indicates its potential for application in neuromorphic computation. The ferroelectricity in HfO_2_ is believed to originate from a noncentrosymmetric orthorhombic phase (o-phase) *Pca*2_1_, formed when a thin HfO_2_ film is crystallized under the appropriate stress and annealing conditions. It has been demonstrated that a tensile in-plane stress in the HfO_2_ induces a transition from the tetragonal phase (t-phase) to the preferred o-phase, thus leading to ferroelectricity.^4^ This is commonly achieved by doping the HfO_2_ and has been accomplished with a wide variety of dopants, with Zr being the most common due to its wide range of doping concentrations, yielding FE properties.^5^ Apart from suitable doping, Böscke et al. highlighted the importance of the top electrode (TE) and its capping ability in achieving the o-phase.^1^ It is believed that similar to the addition of dopants, the TE induces stress on the underlying Hf_1–*x*_Zr_*x*_O_2_ film during annealing. The choice of an appropriate TE can thus enhance the FE properties of HfO_2_. There has since been extensive research exploring various TEs such as Pt,^6^ Mo,^7^ W,^8^ TaN,^9^ and RuO_2_,^10^ but reactively sputtered TiN is prominent.^1,2,5,11^ Even so, reports are scarce on the effect of varying its microstructure. Deposition conditions such as plasma power, pressure, and gas mixture can strongly influence the microstructure of metals deposited by reactive sputtering, which in turn can affect the strain in the film.

In this work, we study the impact of processing conditions for reactively sputtered TiN, when used as a TE for FE Hf_1–*x*_Zr_*x*_O_2_. By employing a combination of electrical characterization and grazing incidence X-ray diffraction (GIXRD), we reveal the importance of (111) textured TiN in achieving FE Hf_1–*x*_Zr_*x*_O_2_. We further use X-ray photoelectron spectroscopy (XPS) and near-edge X-ray absorption fine structure to strengthen our findings. A deepened understanding of the impact of the TE will provide improved reproducibility and performance of HfO_2_-based FE devices.

## Experimental Section

Metal–insulator–semiconductor (MIS) capacitors were fabricated on an InAs(100) substrate (ρ = 3 × 10^–4^ Ω cm) with a 100 nm thick unintentionally n-doped InAs epilayer by metal-organic vapor-phase epitaxy. The native oxide was removed using BOE (1:10) immediately before loading the samples into a Picosun Sunale R-100 atomic layer deposition (ALD) chamber to deposit 12 cycles of Al_2_O_3_, (∼1.2 nm) using TMAl and water as precursors, followed by 100 cycles (∼10 nm) Hf_1–*x*_Zr_*x*_O_2_ deposited by alternating cycles of TEMA(Zr) and TDMA(Hf) in order to achieve a Hf/Zr ratio of 1:1, with water as the oxidizing precursor. All ALD depositions were carried out at a temperature of 200 °C. The use of Al_2_O_3_ as an interfacial layer reduces the native oxide of InAs and decreases interface defect density.^12^ Subsequently, a 10 nm thick TiN TE was deposited using RF magnetron sputtering, with a quartz crystal microbalance thickness meter, to control the deposited layer thickness. The deposition was carried out without a temperature controller at a power of 150 W in an AJA Orion system using a TiN target with 99.5% purity at varying chamber pressures and Ar plasmas. The deposition pressure used was varied in the range 1.3–4.0 mTorr (samples A–C). At a pressure of 4.0 mTorr, two additional samples (samples D–E) were fabricated with the addition of N_2_ into the gas flow during deposition, 6.25 and 12.5%, respectively. The N_2_ gas is inserted at the top of the chamber, in close proximity to the sample. For readability, the samples will be denoted A–E; see deposition conditions summarized in Table 1. After deposition, the samples were annealed using a rapid thermal processing system at 440 °C for 30 s, followed by an additional device pad definition via lift-off of electron beam-evaporated Ti/Pd/Au (1/5/200 nm). Finally, the TiN between devices was removed using a NaOH_4_/H_2_O_2_/H_2_O (1:2:5) wet etch for 30 s at 60 °C.

**Table 1:** Deposition Conditions of TE TiN Samples

sample	pressure [mTorr]	Ar flow [sccm]	N_2_ flow [sccm, (%)]
A	1.3	5	
B	2.6	9	
C	4.0	14	
D	4.0	12	0.75(6.25%)
E	4.0	12	1.5(12.5%)

Electrical characterization was performed using a Keysight B1500A parameter analyzer equipped with a B1530A waveform generator fast measurement unit for pulsed measurements. The conventional positive-up-negative-down (PUND) technique was implemented to measure the polarization versus electric field at a frequency of 1 kHz. The electric field in this paper refers to the electric field applied across the bilayered structure of Al_2_O_3_/Hf_1–*x*_Zr_*x*_O_2_. The PUND measurement was always carried out post wake-up cycling of 1000 times using the same voltage used for the PUND measurement. Cycling measurements were implemented using rectangular pulses at a frequency of 10 kHz at various voltages. For CV measurements, an Agilent 4294A impedance analyzer was used, keeping the oscillation amplitude at 50 mV at frequencies between 10 kHz and 10 MHz. For structural characterization, a Bruker D8 diffractometer with a Cu Kα X-ray source was used for GIXRD measurements with an incidence angle of 0.3–0.5° to determine the crystallographic texturing. A ZEISS Gemini 500 scanning electron microscope with an energy-dispersive X-ray spectroscopy (EDX) detector was used to determine the stoichiometry of the TiN TEs.

## Results and Discussion

**Figure 1:**
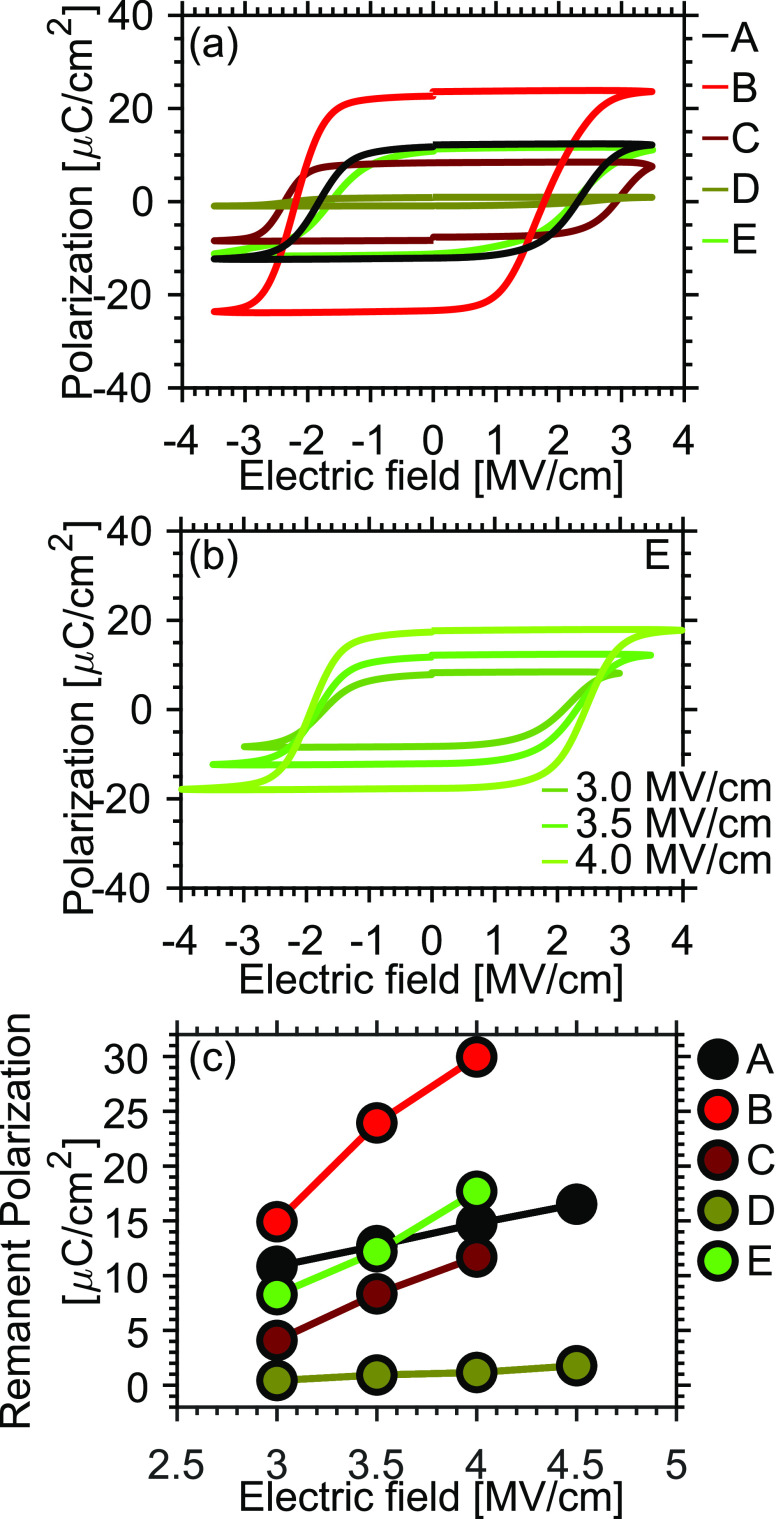
(a) *P*–*E* hysteresis curves at 3.5 V for samples deposited at different pressures and with additional nitrogen, (b) evolution of the PE curve of sample E at increasing electric field, (c) evolution of the remanent polarization *P*_r_^+^ as a function of the applied electric field for all samples.

The polarization–electric field (*P*–*E*) characteristics at 3.5 MV/cm of samples with differently deposited TiN TEs are presented in Figure 1a. The evolution of the *P*–*E* with increasing field for sample E is presented in Figure 1b. The corresponding data and current–voltage characteristics of samples A–E are provided in the Supporting Information, Figure S2. In Figure 1c, the progression of the remanent polarization at increasing electric field of all samples is summarized. The *P*_r_ values in Figure 1c are extracted from the *P*–*E* curves presented in the Supporting Information, Figure S2. The observed leakage currents are much smaller than the switching currents for all the samples, facilitating precise extraction of *P*–*E* characteristics (Supporting Information, Figure S1). From this data, the crucial impact of the TE deposition conditions is evident. Sample A with a TE deposited at the lowest deposition pressure of 1.3 mTorr exhibits a remanent polarization of 14 μC/cm^2^ at a maximum electric field of 4 MV/cm (Figure 1c). Increasing the pressure to 2.6 mTorr (sample B) leads to a strong improvement of *P*_r_ up to 30 μC/cm^2^. However, further increasing the chamber pressure during TiN deposition (sample C) severely decreases *P*_r_ again to 10 μC/cm^2^. Interestingly, upon the addition of a low N_2_ flow of 6.25% at 4.0 mTorr deposition pressure, the polarization almost vanishes (sample D). Furthermore, with the N_2_ flow increased to 12.5%, the magnitude of *P*_r_ increases again to 17 μC/cm^2^ (sample E).

**Figure 2:**
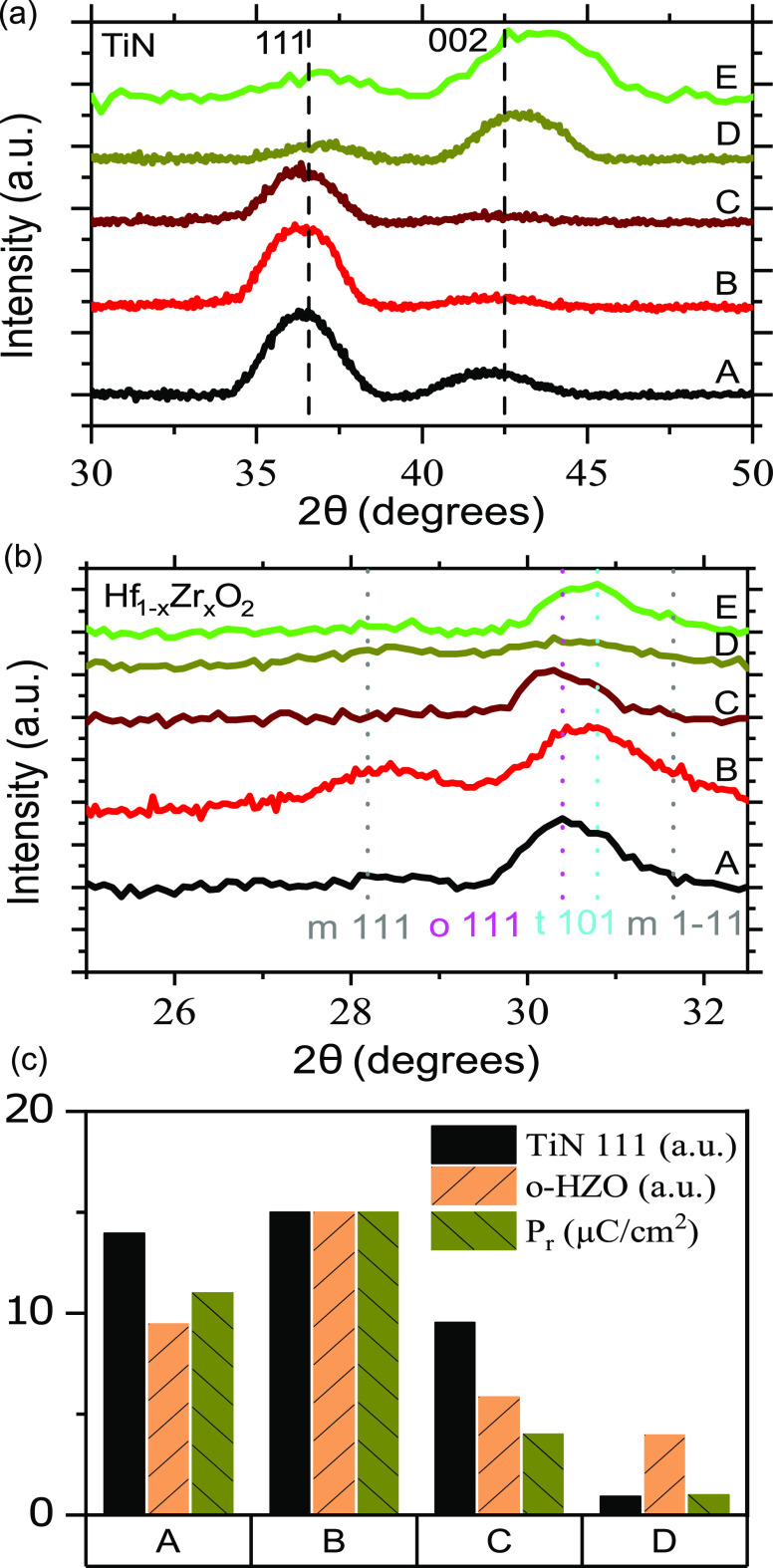
(a) GIXRD of TiN on silicon between 30 and 50° with dashed vertical lines indicating the position of the (111) and (002) reflection from unstrained TiN, (b) GIXRD of Hf_1–*x*_Zr_*x*_O_2_ between 25 and 33°. Positions of the monoclinic m-(111), o-(111), t-(101), and m-(−1–11) reflections are indicated. (c) Correlation between TiN(111) texturing, Hf_1–*x*_Zr_*x*_O_2_ o-(111) phase volumetric fraction and the remanent polarization *P*_r_.

To gain insights into the large differences in the electrical data for the various TEs their crystal structure was investigated by means of GIXRD. Figure 2a compares the measured reflections of TiN films deposited directly on Si substrates under the same conditions as used for the MIS samples (see Table 1). Sputtered TiN tends to have a preferential grain orientation (texturing) either with (111) or (002) aligned to the sample surface normal depending on the processing conditions.^13^ The (111) and (002) reflections for cubic TiN are found at 2θ = 36.5° and 2θ = 42.5°, respectively.^14^ The measured data in Figure 2a indicate that both (111) and (002) reflections are present in the films to different extents depending on the sputtering parameters used. Due to the limited diffraction signal of thin polycrystalline films in the regular Bragg geometry, we are forced to utilize GIXRD geometry here, in which the measured (111) and (002) planes are inclined at 18.25 and 21.25° with respect to the surface normal. The similar inclinations of the two reflections allow us to obtain an indication of the preferential grain orientation from the relative peak intensities. A clear trend is observed with increasing chamber pressure. For sample A deposited with the lowest pressure of 1.3 mTorr, both (111) and (002) grain orientations are present. However, when the pressure is increased to 2.6 mTorr (sample B), the (002) reflection is reduced. Finally, for sample C with the highest deposition pressure of 4.0 mTorr, there is no longer a distinct reflection from (002) oriented grains, and the (111) reflection is lower in intensity compared to A and B, indicating lower degree of overall TiN crystallinity. This corresponds well with previously reported trends for TiN sputtering in literature and can be explained by a decreased ion energy with higher deposition pressures.^13^ The preferred texturing of TiN is decided by overall energy minimization, which for TiN becomes a competition between the low strain energy of the (111) plane and low surface energy of the (002) plane.^15^ At low ion energy (high pressure), the strain energy dominates and (111) texturing is thus preferred, while at higher ion energy (low pressure), surface energy dominates, favoring (002) texturing.^13^ Interestingly, for samples D and E where a N_2_ flow is introduced, the (002) reflection is predominant. To understand this, it is important to note that the TiN (111) plane is fully nitrogen-terminated, whereas the (002) plane is stoichiometric. With the addition of N_2_ flow during deposition we obtain a surplus of N adatoms at the surface, which strongly decreases the mean free path of Ti adatoms on the (001) surface and promotes the growth on these planes, resulting in a preferred (002) texturing.^13^

GIXRD data of the Hf_1–*x*_Zr_*x*_O_2_ layer in the MIS samples after annealing, presented in Figure 2b, highlights the impact of the TiN texturing. The desired FE o-(111) reflection is expected at 2θ = 30.4°,^16^ whereas the non-FE, m-(111), is found at 2θ = 28°. A clear trend between predominately TiN (111) texturing and the intensity of the FE o-(111) reflection of the Hf_1–*x*_Zr_*x*_O_2_ can be observed. For sample D with a low N_2_ flow and predominantly (002) textured TiN, the lowest intensity of the o-(111) reflection is measured. Overall, the crystallinity is weak in this sample. On the contrary, sample B with (111) textured TiN has the strongest o-(111) reflection. As is typically reported for Hf_1–*x*_Zr_*x*_O_2_,^1^ there is a strong correlation between the peak height of the o-(111) reflection and the remanent polarization *P*_r_ magnitude. By accounting for the TiN TE, we provide further insights, revealing the additional interplay between the strength of the TiN (111) texturing and the strength of the Hf_1–*x*_Zr_*x*_O_2_ o-(111) reflection. This correlation is highlighted in Figure 2c. For information regarding the calculation of the relative peak intensities, we refer to the Supporting Information.

It is important to understand why the conditions yielding predominant (111) texturing of the TiN also lead to a strong FE response of the Hf_1–*x*_Zr_*x*_O_2_ film, while the FE response diminishes with stronger (002) texturing. A possible explanation for this lies in the induced strain from the TiN on the Hf_1–*x*_Zr_*x*_O_2_ during the crystallization process. For TiN with (002) texturing, there will be compressive stress (up to −0.7 GPa) acting on the underlying Hf_1–*x*_Zr_*x*_O_2_, whereas in the case of (111) texturing, a tensile stress (up to 1.4 GPa) is present instead.^13^ Indeed, we here extract a ∼1% out-of-plane tensile strain from the TiN peak positions in samples A–C, confirmed by wafer curvature measurements. In samples D and E, we instead observe ∼1% compressive strain (Supporting Information, Figures S3 and S4). Thus for samples A–C, we expect the out-of-plane tensile strain in the TiN to induce an in-plane tensile stress in the underlying Hf_1–*x*_Zr_*x*_O_2_ layer. There are many reports of in-plane tensile stress enhancing the out-of-plane FE properties since it allows for the transformation between the *c*-axis of the tetragonal phase into the *a*-axis of the orthorhombic FE phase.^4,16−20^ It is worth noting that despite having predominantly (002) texturing, sample E with TiN deposited with 12.5% N_2_ exhibits a relatively strong o-(111) reflection and in turn, a *P*_r_ = 17 μC/cm^2^ at 4 V bias. Clearly, for this sample, the TiN texturing cannot explain the presence of the FE Hf_1–*x*_Zr_*x*_O_2_ phase and has therefore been excluded in Figure 2c. We believe that the origin of *P*_r_ in sample E is due to the high level of added nitrogen during deposition of TiN, as was previously observed by Luo et al.^11^

**Figure 3:**
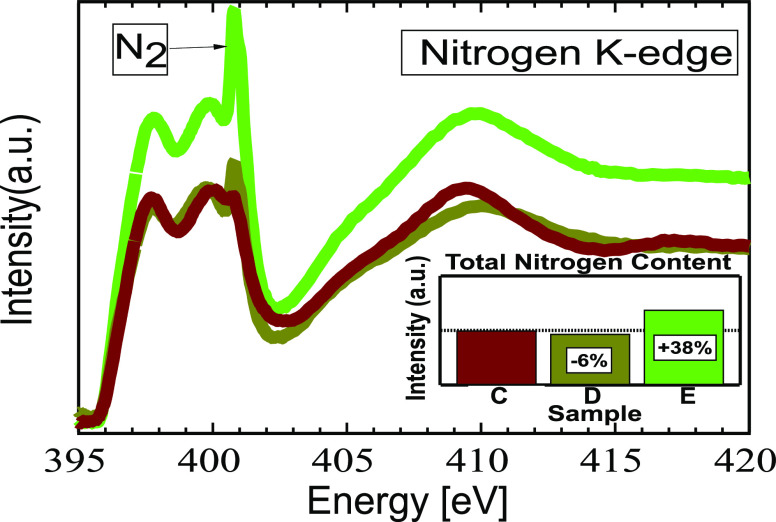
XAS and integrated intensity of nitrogen K-edges. Spectra are of TiN deposited with (C) 0% N_2_, 100% Ar flow (dark red), (D) 6.25% N_2_ flow (dark green), and (E) 12.5% N_2_ flow (bright green).

Complementing the electrical measurements, NEXAFS and XPS were conducted to investigate compositional differences between samples. MIS structures with 2 nm thin TiN layers were processed using the same deposition conditions as before (see Table 1) to accommodate the surface sensitivity of these techniques. Figure 3 shows X-ray absorption spectra (XAS) comparing the nitrogen K-edges of samples with varying N_2_ gas flows. Samples D (dark green, 6.25% N_2_ flow) and E (bright green, 12.5% N_2_ flow) are juxtaposed with nitrogen-free deposition, sample C (dark red, 100% Ar flow). Increasing the nitrogen flow increased the sharp, high-energy shoulder at 400.8 eV, which is characteristic of the N 1s → 1π* transition of molecular nitrogen.^21^ It is noted that this signal was present even in sample C without N_2_ flow, evidence of a byproduct of oxidation. Nonetheless, the peak size was concomitant with flow, indicating that nitrogen gas had incorporated into the samples. This is reinforced by high-resolution NEXAFS spectra which exhibit the vibrational characteristics of N_2_ trapped in lattice interstices^22^ (Supporting Information, Figure S5). The molecular nitrogen peaks were diminished in the more surface-sensitive partial electron yield NEXAFS and absent from XPS, indicating that N_2_ was mainly incorporated close to or even in the Hf_1–*x*_Zr_*x*_O_2_ layer, not in the strongly oxidized surface of the TiN layer. The inset of Figure 3 quantifies a more salient point; the entire N K-edge was integrated to get an estimate of the total nitrogen content. Whereas increasing gas flow from 0 to 6.25% N_2_ resulted in approximately the same amount of nitrogen signal (samples C, D), further increasing it to 12.5% (sample E) came with a nearly 40% increase in nitrogen. Combined with the diffraction data, we interpret this as clear evidence of added N_2_-gas flow changing the TiN chemical composition and even introducing molecular N_2_ into the Hf_1–*x*_Zr_*x*_O_2_. As was previously hypothesized,^11^ interstitial N_2_ may induce the necessary stress to facilitate the phase transition into the FE phase, explaining the high *P*_r_ observed in sample E, despite having predominately (002) TiN texturing.

**Figure 4:**
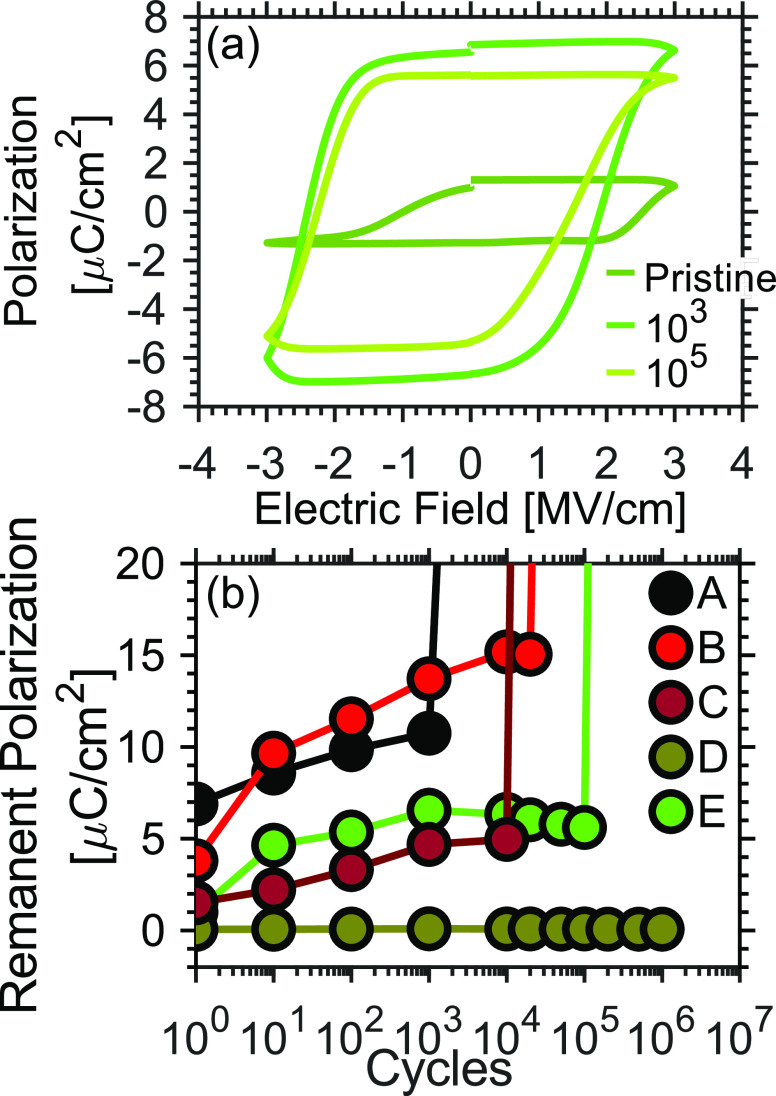
(a) Evolution of the PE curve of sample E at 3 V during cycling from pristine to 10^5^ cycles and (b) cycling endurance at 3 V at a frequency of 10 kHz displaying the changes in the remanent polarization *P*_r_ as a function of switching cycles.

Next, we evaluate the impact of the TE processing conditions on the cycling endurance of the Hf_1–*x*_Zr_*x*_O_2_ films. In Figure 4a, we present the *P*–*E* characteristics of sample E in its pristine state after 10^3^ and 10^5^ cycles. Corresponding measurements were done for samples A–D, where the evolution of the *P*_r_ with cycling is presented in Figure 4b. The endurance data were measured by cycling rectangular pulses with an amplitude of 3 V at a frequency of 10 kHz. The polarization was measured with the PUND technique at a frequency of 1 kHz once per decade up to 10^4^ cycles, followed by more frequent measurements up to 10^6^ cycles. It is clear that the “wake-up” effect in the samples is different, possibly due to variations in the oxygen vacancy distribution. Three devices of each sample were measured, and similar results were achieved for all devices of a sample. In general, we observe that the endurance of the samples is limited by the InAs bottom electrode, known for a large interfacial defect concentration^23^ (Supporting Information, Figure S6). Here, we focus on the pronounced relative differences between the samples. Samples A and C with TiN deposited at 1.3 and 4.0 mTorr both break down earlier (10^3^ and 10^4^ cycles, respectively) than sample B with TiN deposited at 2.6 mTorr (2 × 10^4^ cycles). Importantly, sample B has both the highest polarization of the three and superior endurance properties. Adding N_2_ during TiN deposition appears to improve the endurance properties of the Hf_1–*x*_Zr_*x*_O_2_; sample D handles more than the maximum 10^6^ cycles of the experiment, while sample E breaks down at 10^5^ cycles, five times higher than the best devices with TiN deposited without N_2_. It is noteworthy that there does not seem to be a correlation between the remanent polarization and the endurance of the samples. In other words, a high *P*_r_ does not necessarily reduce endurance, despite the strong internal fields arising due to the remanent polarization. Still, the superior endurance of sample D could be explained by the combination of added N_2_ together with a very low remanent polarization.

It is a common explanation that one of the limiting factors for cycling endurance in Hf_1–*x*_Zr_*x*_O_2_-based FE devices is generation and accumulation of oxygen vacancy defects, which ultimately lead to hard breakdown of the oxide.^24^ Titanium is a known oxygen scavenger which drives this effect.^25^ Thus, N deficiencies in the TiN TE may negatively influence the FE endurance by scavenging oxygen from the Hf_1–*x*_Zr_*x*_O_2_. In support of this, Lin et al. recently indicated an increase of oxygen vacancies together with the breakage of Hf–O bonds when exposing a TiN/HfO_2_ structure to electrical stress.^26^ It can therefore be beneficial to deposit a N-rich TiN TE to minimize the oxygen scavenging effect, especially when endurance is of importance. Thus, we propose that the extended endurance observed with added N_2_ during TiN deposition is a result of increased nitrogen incorporation in the TiN film, leading to less oxygen scavenging. EDX analysis supports this, as a higher nitrogen content was found for samples D (57.2 at. %) and E (59.3 at. %) when compared to sample C (55.1 at. %) (Supporting Information, Table S1 and Figure S7). Moreover, XPS data from samples C, D, and E corroborate the observations, showing increased nitrogen incorporation with gas flow. The Ti 2p core level was monitored for chemical shifts over an incident X-ray energy varying from 550 to 1150 eV, corresponding to an inelastic mean free path of the photoemitted electrons that was sufficient for characterizing the top 1–2 nm (Supporting Information, Figure S8). Although significant oxidation of TiN occurred upon exposure to ambient air, titanium oxide and oxynitride components were deconvoluted and their relative components quantified. The addition of high levels of nitrogen during TiN deposition (sample E) led to more (and presumably more thermodynamically stable) Ti–N species, which were less prone to oxidation. Supporting details are summarized in Table S2 and Figure S9.

**Figure 5:**
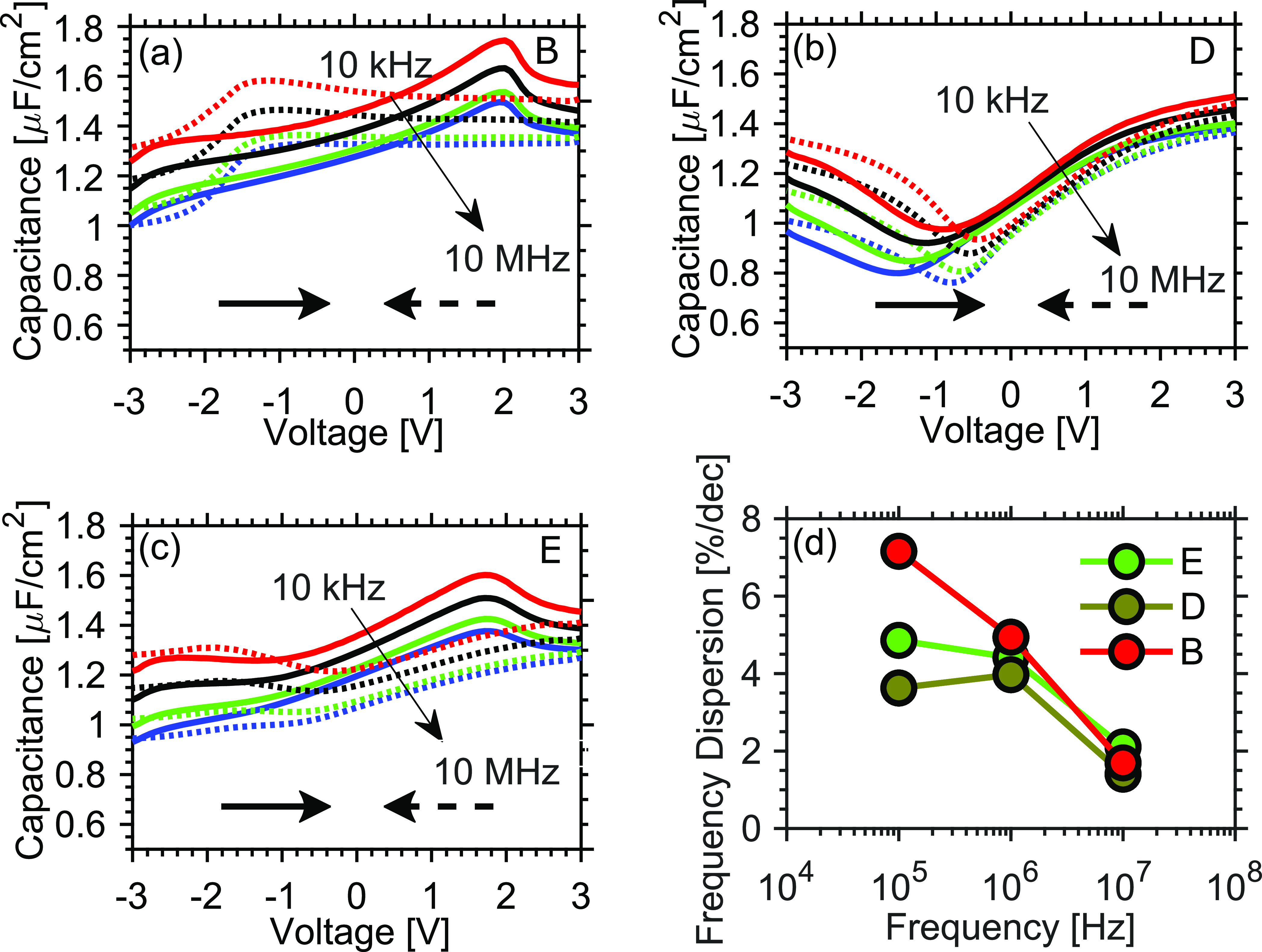
Capacitance–voltage characteristics between −3 and 3 V for frequencies between 10 kHz and 10 MHz for samples B (a), D (b) and E (c). The arrows below the curves indicate the sweeping direction of the measurement. In (d), the frequency dispersion per decade of the corresponding samples is presented.

To further explore the potential accumulation of defects in the Hf_1–*x*_Zr_*x*_O_2_ upon voltage cycling, capacitance–voltage (CV) characteristics were investigated. In Figure 5a–c, the measured CV data for samples B, D, and E are presented, where the bias is first swept from −3 to 3 V (solid) and then back to −3 V (dotted) while measuring the small signal response with an AC signal of varying frequency and amplitude of 50 mV. The frequency dispersion per decade in accumulation is investigated between the three samples in Figure 5d. Sample B in Figure 5a behaves as expected for a regular MIS structure with high capacitance when the semiconductor is in accumulation (>1 V) and by applying a negative voltage to the top contact the InAs is driven into depletion, leading to a decreased capacitance. Due to thermally excited minority carriers, there is an increase in capacitance again once the bias is reduced below −1 V, which is typical at room temperature for narrow band gap III–V materials such as InAs.^27−29^ As the frequency is increased, the capacitive contributions from minority carriers and defect states are reduced. For both samples B (Figure 5a) and E (Figure 5c), the typical “butterfly”-shaped CV curves are measured, exhibiting two distinct peaks arising from the increased capacitance during polarization switching. From the CV characteristics of the samples, the frequency dispersion per decade in accumulation can be extracted (Figure 5d). The dispersion between measurement frequencies allows one to qualitatively compare the distribution of defects in the dielectric from their time constants. From Figure 5d, it becomes evident that the introduction of N_2_ during deposition results in a smaller frequency dispersion per decade, with both samples D and E having lower dispersion up to 1 MHz. These results could indicate that a N_2_-rich TiN reduces oxygen vacancy defects in the Hf_1–*x*_Zr_*x*_O_2_ film, which reduces the capacitive influence of defect states. At the higher frequencies of 1–10 MHz, the dispersion becomes similar for all three samples. At these high frequencies, only defects with short tunneling lengths can respond. Hence, the contribution becomes dominated by defects located close to the semiconductor interface, which in this case would originate from the thin Al_2_O_*x*_ interface layer for all the measured samples.

## Conclusions

This paper highlights the importance of the deposition conditions for sputtered TiN when used as a TE for FE Hf_1–*x*_Zr_*x*_O_2_. It is found that the remanent polarization of Hf_1–*x*_Zr_*x*_O_2_ films is heavily impacted by the texturing of the TiN film, ranging from almost zero for majority (002) texture up to 30 μC/cm^2^ for the majority (111) texture. Moreover, additional N_2_ flow during TiN sputtering yields improved endurance and a lower CV frequency dispersion. XAS, EDX, and XPS measurements confirm the increased nitrogen incorporation in the TiN with additional N_2_ flow. Finally, we hypothesize that the disparate results in the literature, with regard to the magnitude of remanent polarization, ranging from 10^30^ to 26 μC/cm^2^,^31^ obtained with essentially the same material stack, could be a result of varying processing conditions for the TiN electrodes, rather than differences of Hf_1–*x*_Zr_*x*_O_2_ itself.

## Supporting Information Available

Measured current voltage characteristics of PUND measurements and corresponding *P*–*V* and *I*–*V* curves of samples A–E; extraction procedure of bar height in Figure 2c; microstrain calculations derived from the measured GIXRD data of the TiN(111) and (002) reflection; stress determination by wafer curvature measurements; vibrational oscillations of molecular nitrogen in NEXAFS results; cycling endurance comparison between MIM and MIS structures (sample B); relative compositional changes of the TiN TE in samples C–E derived from the EDX data; and deconvolution of the XPS data and relative peak components of samples C–E for Ti 2p (PDF)

## References

[ref1] Böscke T. S.; Müller J.; Bräuhaus D.; Schröder U.; Böttger U. Ferroelectricity in Hafnium Oxide Thin Films. Appl. Phys. Lett. 2011, 99, 102903 10.1063/1.3634052.

[ref2] Mikheev V.; Chouprik A.; Lebedinskii Y.; Zarubin S.; Matveyev Y.; Kondratyuk E.; Kozodaev M. G.; Markeev A. M.; Zenkevich A.; Negrov D. Ferroelectric Second-Order Memristor. ACS Appl. Mater. Interfaces 2019, 11, 32108–32114. 10.1021/acsami.9b08189. 31402643

[ref3] Oh S.; Kim T.; Kwak M.; Song J.; Woo J.; Jeon S.; Yoo I. K.; Hwang H. HfZrOx-Based Ferroelectric Synapse Device with 32 Levels of Conductance States for Neuromorphic Applications. IEEE Electron Device Lett. 2017, 38, 732–735. 10.1109/led.2017.2698083.

[ref4] Shiraishi T.; Katayama K.; Yokouchi T.; Shimizu T.; Oikawa T.; Sakata O.; Uchida H.; Imai Y.; Kiguchi T.; Konno T. J.; Funakubo H. Impact of Mechanical Stress on Ferroelectricity in (Hf0.5Zr0.5)O2 Thin Films. Appl. Phys. Lett. 2016, 108, 262904 10.1063/1.4954942.

[ref5] Müller J.; Böscke T. S.; Schröder U.; Mueller S.; Bräuhaus D.; Böttger U.; Frey L.; Mikolajick T. Ferroelectricity in Simple Binary ZrO 2 and HfO 2. Nano Lett. 2012, 12, 4318–4323. 10.1021/nl302049k. 22812909

[ref6] Lin Y.-C.; McGuire F.; Franklin A. D. Realizing Ferroelectric Hf 0.5 Zr 0.5 O 2 with Elemental Capping Layers. J. Vac. Sci. Technol., B: Nanotechnol. Microelectron.: Mater., Process., Meas., Phenom. 2018, 36, 011204 10.1116/1.5002558.

[ref7] Chen K. T.; Liao C. Y.; Lo C.; Chen H. Y.; Siang G. Y.; Liu S.; Chang S. C.; Liao M. H.; Chang S. T.; Lee M. H. Improvement on Ferroelectricity and Endurance of Ultra-Thin HfZrO2 Capacitor with Molybdenum Capping Electrode. 2019 Electron Devices Technology and Manufacturing Conference, EDTM 2019, 2019; pp 62–64.

[ref8] Karbasian G.; Dos Reis R.; Yadav A. K.; Tan A. J.; Hu C.; Salahuddin S. Stabilization of Ferroelectric Phase in Tungsten Capped Hf0.8Zr0.2O2. Appl. Phys. Lett. 2017, 111, 022907 10.1063/1.4993739.

[ref9] Lomenzo P. D.; Takmeel Q.; Zhou C.; Fancher C. M.; Lambers E.; Rudawski N. G.; Jones J. L.; Moghaddam S.; Nishida T. TaN Interface Properties and Electric Field Cycling Effects on Ferroelectric Si-Doped HfO2 Thin Films. J. Appl. Phys. 2015, 117, 134105 10.1063/1.4916715.

[ref10] Park M. H.; Kim H. J.; Kim Y. J.; Jeon W.; Moon T.; Hwang C. S. Ferroelectric Properties and Switching Endurance of Hf0.5Zr0.5O2 Films on TiN Bottom and TiN or RuO2 Top Electrodes. Phys. Status Solidi RRL 2014, 8, 532–535. 10.1002/pssr.201409017.

[ref11] Luo J.-D.; Yeh Y.-T.; Lai Y.-Y.; Wu C.-F.; Chung H.-T.; Li Y.-S.; Chuang K.-C.; Li W.-S.; Chen P.-G.; Lee M.-H.; Cheng H.-C. Correlation between Ferroelectricity and Nitrogen Incorporation of Undoped Hafnium Dioxide Thin Films. Vacuum 2020, 176, 109317 10.1016/j.vacuum.2020.109317.

[ref12] Timm R.; Fian A.; Hjort M.; Thelander C.; Lind E.; Andersen J. N.; Wernersson L. E.; Mikkelsen A. Reduction of Native Oxides on InAs by Atomic Layer Deposited Al 2O3 and HfO2. Appl. Phys. Lett. 2010, 97, 132904 10.1063/1.3495776.

[ref13] Petrov I.; Barna P. B.; Hultman L.; Greene J. E. Microstructural Evolution during Film Growth. J. Vac. Sci. Technol., A 2003, 21, S117–S128. 10.1116/1.1601610.

[ref14] Wu H. Z.; Chou T. C.; Mishra A.; Anderson D. R.; Lampert J. K.; Gujrathi S. C. Characterization of Titanium Nitride Thin Films. Thin Solid Films 1990, 191, 55–67. 10.1016/0040-6090(90)90274-h.

[ref15] Jafarzadeh M.; Khojier K.; Savaloni H. Influence of Nitrogen Gas Flow on Mechanical and Tribological Properties of Sputtered Chromium Nitride Thin Films. Adv. Mater. Res. 2014, 829, 497–501. 10.4028/www.scientific.net/AMR.829.497.

[ref16] Hyuk Park M.; Joon Kim H.; Jin Kim Y.; Lee W.; Moon T.; Seong Hwang C. Evolution of Phases and Ferroelectric Properties of Thin Hf 0.5Zr0.5O2 Films According to the Thickness and Annealing Temperature. Appl. Phys. Lett. 2013, 102, 242905 10.1063/1.4811483.

[ref17] Huan T. D.; Sharma V.; Rossetti G. A.; Ramprasad R. Pathways towards Ferroelectricity in Hafnia. Phys. Rev. B: Condens. Matter Mater. Phys. 2014, 90, 064111 10.1103/physrevb.90.064111.

[ref18] Kisi E. H. Influence of Hydrostatic Pressure on the t → o Transformation in Mg-PSZ Studied by in Situ Neutron Diffraction. J. Am. Ceram. Soc. 1998, 81, 741–745. 10.1111/j.1151-2916.1998.tb02402.x.

[ref19] Lowther J. E.; Dewhurst J. K.; Leger J. M.; Haines J. Relative Stability of ZrO2 and HfO2 Structural Phases. Phys. Rev. B: Condens. Matter Mater. Phys. 1999, 60, 14485–14488. 10.1103/physrevb.60.14485.

[ref20] Park M. H.; Kim H. J.; Kim Y. J.; Moon T.; Hwang C. S. The Effects of Crystallographic Orientation and Strain of Thin Hf 0.5Zr0.5O2 Film on Its Ferroelectricity. Appl. Phys. Lett. 2014, 104, 072901 10.1063/1.4866008.

[ref21] Chen C. T.; Ma Y.; Sette F. K-Shell Photoabsorption of the N2 Molecule. Phys. Rev. A: At., Mol., Opt. Phys. 1989, 40, 6737–6740. 10.1103/physreva.40.6737. 9902078

[ref22] Petravic M.; Gao Q.; Llewellyn D.; Deenapanray P. N. K.; Macdonald D.; Crotti C. Broadening of Vibrational Levels in X-Ray Absorption Spectroscopy of Molecular Nitrogen in Compound Semiconductors. Chem. Phys. Lett. 2006, 425, 262–266. 10.1016/j.cplett.2006.05.056.

[ref23] Babadi A. S.; Lind E.; Wernersson L. E. Modeling of N-InAs Metal Oxide Semiconductor Capacitors with High-κ Gate Dielectric. J. Appl. Phys. 2014, 116, 214508 10.1063/1.4903520.

[ref24] Bersuker G.; Heh D.; Young C.; Park H.; Khanal P.; Larcher L.; Padovani A.; Lenahan P.; Ryan J.; Lee B. H.; Tseng H.; Jammy R. Breakdown in the Metal/High-k Gate Stack: Identifying the “Weak Link” in the Multilayer Dielectric. Technical Digest—International Electron Devices Meeting; IEDM, 2008; pp 3–6.

[ref25] Filatova E. O.; Sakhonenkov S. S.; Konashuk A. S.; Kasatikov S. A.; Afanas’ev V. V. Inhibition of Oxygen Scavenging by TiN at the TiN/SiO2 Interface by Atomic-Layer-Deposited Al2O3 Protective Interlayer. J. Phys. Chem. C 2019, 123, 22335–22344. 10.1021/acs.jpcc.9b05800.

[ref26] Lin Y.-J.; Teng C.-Y.; Chang S.-J.; Liao Y.-F.; Hu C.; Su C.-J.; Tseng Y.-C. Role of Electrode-Induced Oxygen Vacancies in Regulating Polarization Wake-up in Ferroelectric Capacitors. Appl. Surf. Sci. 2020, 528, 147014 10.1016/j.apsusc.2020.147014.

[ref27] Babadi A. S.; Lind E.; Wernersson L.-E. ZrO2 and HfO2 Dielectrics on (001) n-InAs with Atomic-Layer-Deposited in Situ Surface Treatment. Appl. Phys. Lett. 2016, 108, 132904 10.1063/1.4945430.

[ref28] Persson A. E. O.; Athle R.; Littow P.; Persson K. M.; Svensson J.; Borg M.; Wernersson L. E. Reduced Annealing Temperature for Ferroelectric HZO on InAs with Enhanced Polarization. Appl. Phys. Lett. 2020, 116, 062902 10.1063/1.5141403.

[ref29] Persson A. E. O.; Athle R.; Svensson J.; Borg M.; Wernersson L.-E. A Method for Estimating Defects in Ferroelectric Thin Film MOSCAPs. Appl. Phys. Lett. 2020, 117, 242902 10.1063/5.0029210.

[ref30] Lee Y. H.; Kim H. J.; Moon T.; Kim K. D.; Hyun S. D.; Park H. W.; Lee Y. B.; Park M. H.; Hwang C. S. Preparation and Characterization of Ferroelectric Hf0.5Zr0.5O2 Thin Films Grown by Reactive Sputtering. Nanotechnology 2017, 28, 305703 10.1088/1361-6528/aa7624. 28562366

[ref31] Kim S. J.; Narayan D.; Lee J.-G.; Mohan J.; Lee J. S.; Lee J.; Kim H. S.; Byun Y. C.; Lucero A. T.; Young C. D.; Summerfelt S. R.; San T.; Colombo L.; Kim J. Large Ferroelectric Polarization of TiN/Hf0.5Zr0.5O2/TiN Capacitors Due to Stress-Induced Crystallization at Low Thermal Budget. Appl. Phys. Lett. 2017, 111, 242901 10.1063/1.4995619.

