# Supplementary material for: Effects of TiN Top Electrode Texturing on Ferroelectricity in Hf1–xZrxO2
Source: ACS Appl Mater Interfaces. 2021 Feb 24;13(9):11089–95. doi: 10.1021/acsami.1c01734 (PMC8027987; doi:10.1021/acsami.1c01734)
Supplement: Supplementary file 1 — am1c01734_si_001.pdf [file am1c01734_si_001.pdf]

Supporting Information

Effects of TiN Top Electrode Texturing on Ferroelectricity in $Hf_{1-x}Zr_xO_2$.

Robin Athle[1, 3], Anton E. O. Persson[1], Austin Irish[2, 3], Heera Menon[1, 3], Rainer Timm[2, 3] and Mattias Borg[1, 3]*

[1] Electrical and Information Technology, Lund University, Box 118, 22 100 Lund, Sweden

[2] Division of Synchrotron Radiation Research, Lund University, Box 118, 22 100 Lund, Sweden

[3] NanoLund Lund University, Box 118, 22 100 Lund, Sweden

Corresponding Author: robin.atle@eit.lth.se

Measured current voltage characteristics during PUND

The measured current and voltage characteristics of the PUND measurements is provided for all samples in **Figure S1** below, with the purpose of ruling out any impact of the leakage current in the extraction of the remanent polarization values provided in the main manuscript.

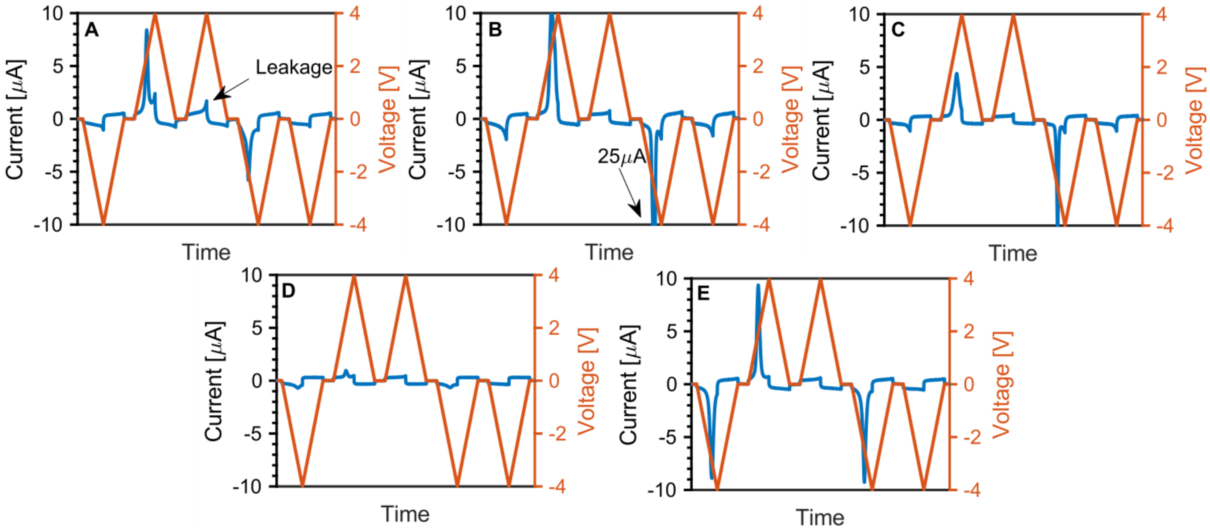

Figure S1: Measured current in the PUND method for correspondingly applied voltages for samples A-E, highlighting the low leakage current at the highest electric field of 4.0 MV/cm.

P-V and I-V characteristics of PUND measurements

Figure S2 shows the P-V and I-V curves of samples A-E from where the remanent polarization values in Figure 1(c) was extracted.

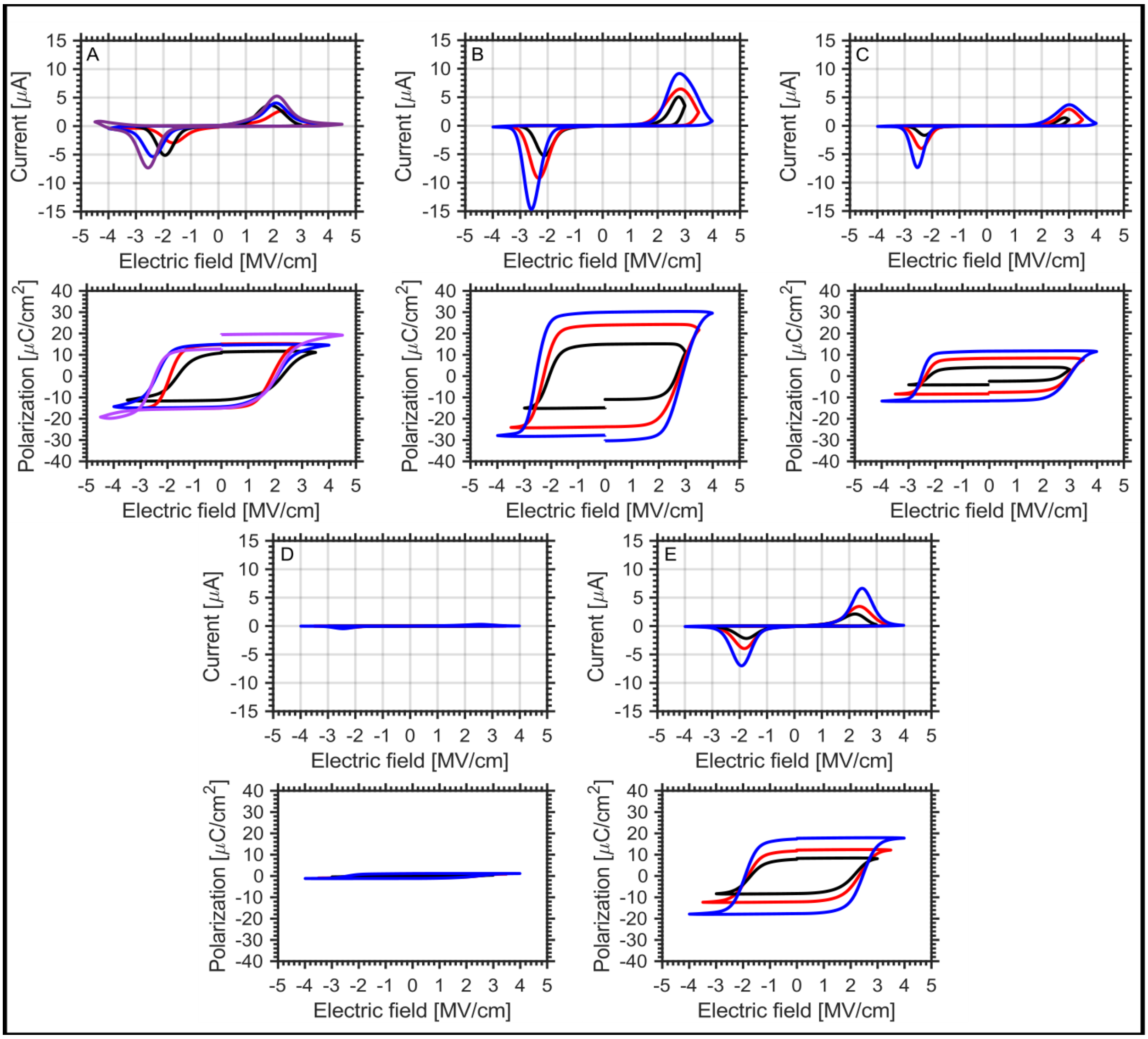

Figure S2: P-V and I-V curves of samples A-E at 3 MV/cm (black), 3.5 MV/cm (red), 4 MV/cm (blue) and 4.5 MV/cm (purple).

Calculation of bar height in Figure 2(c)

The aim of Figure 2(c) is to visualize the correlation between TiN (111) texturing, amount of o-phase $Hf_xZr_{1-x}O_2$ and the remanent polarization measured in the capacitors. To condense this correlation into three comparable numbers we did the following:

c-TiN (111): We measured the areas under the c-TiN (111) peak (A_{111}) and c-TiN (002) peak (A_{002}), respectively. Then compare these areas to get an appreciation of the degree of (111) texturing for the given sample. We take into account that the (111) reflection is theoretically 67% as strong as the (002) reflection for TiN by multiplying the measured area for the (111) reflection by 1/0.67. Thus, the fraction of (111) texturing can be expressed as follows:

$$X_{111} = \frac{A_{111}}{0.67\left(\frac{A_{111}}{0.67} + A_{002}\right)} \quad (1)$$

To take into account the absolute amount of crystallization in the TiN film we multiply X_{111} by A_{111}, as the overall signal will depend on the degree of crystallization. We finally normalize the bar height so that the highest bar matches the highest $P_r = 15\ \mu C/cm^2$ so that they are easily comparable. This then gives us the bar height as

$$H_{TiN} = X_{111}A_{111}\frac{\max(P_r)}{\max(X_{111}A_{111})} \quad (2)$$

It should be noted that all TiN films were deposited on 52 mm (2 inch) diameter Si wafers which are much larger than the expected x-ray footprint at 0.5° incidence angle with a 0.1 mm incidence slit (footprint ~11 mm). Thus, we are confident that the absolute intensities can indeed be compared between samples in this way.

o-HZO (111): Here we measure the area under the o-$Hf_xZr_{1-x}O_2$ (111) peak (A_{o111}) and normalize it in a similar way as for TiN so that the highest bar matches the maximum P_r for easier comparison. Thus, the bar height is given by

$$H_{HZO} = A_{o111}\frac{\max(P_r)}{\max(A_{o111})} \quad (3)$$

P_r: Remanent polarization P_r in the unit $\mu C/cm^2$ was measured for samples corresponding to the TiN deposition conditions of A-D, and we directly use these values for the bar height. Thus, the bar height is given by:

$$H_{Pr} = P_r \quad (4)$$

Strain Calculations from XRD Data

Using the shifts away from the theoretical positions of the reflections, we can estimate the strain (ε) in the TiN films. We have done this for both the (111) and (002) reflectionsfor samples A-E, which is presented in **Figure S3**. The strain was calculated by first calculating the lattice spacing given by the peak position according to Bragg's law, here shown for the example of the (111) reflection:

$$d_{111} = \frac{\lambda}{2\sin\theta} \quad (5)$$

Where λ is the wavelength of the x-rays (Cu Kα → 0.154 nm), and θ is the angle of reflection. The strain is then given by $\varepsilon = \frac{d_{111} - d_{111}^0}{d_{111}^0}$, where $d_{111}^0 = 0.245\ nm$ is the tabulated unstrained lattice constant of the reflection. A similar analysis was done for the (002) reflection, with $d_{002}^0 = 0.212\ nm$. The results for both the (111) and (002) reflections are presented in **Figure S3**, where it is evident that for samples A-C there is up to 1% tensile strain in the TiN film. While for samples D and E the strain instead becomes compressive, for both the (111) and (002) reflection, all in accordance with what is expected from literature[1].

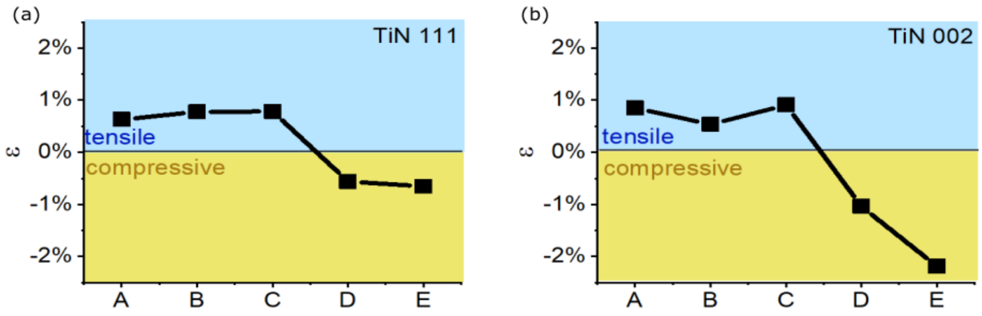

Figure S3: Calculated micro-strain of samples A-E from the (a) TiN (111) and (b) TiN (002) reflections.

Stress Wafer Curvature Measurement

To complement the stress/strain analysis derived from the GIXRD wafer curvature measurements were conducted for samples A-C. Through implementation of the Stoney equation [2] the stress in the deposited TiN films were calculated with the results presented in **Figure S4**. For all the samples a high compressive stress of roughly 1 GPa and above is measured. Sample A has the highest compressive stress of -3.5 GPa whereas in samples B and C the stress values are comparable around -1 GPa. In-plane compressive stress leads to out-of-plane tensile stress, which is in line with the observations by GIXRD for samples A-C.

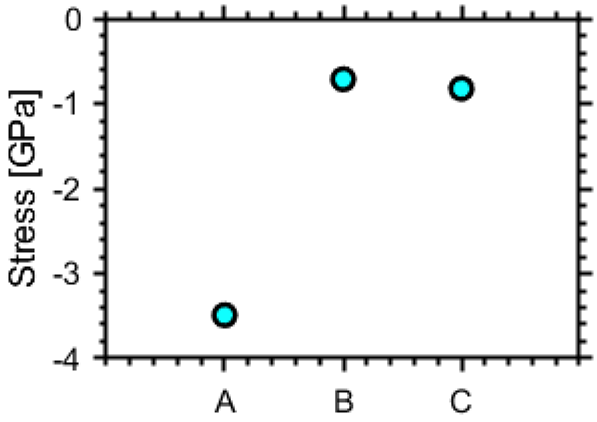

Figure S4. The calculated stress in TiN deposited with the processing conditions of samples A-C.

NEXAFS of vibrational oscillations of molecular nitrogen

Figure S5 resolves the molecular nitrogen peaks observed at 400.8 eV, which are characteristic of interstitially trapped nitrogen gas. The first vibrational state, ν_{00} = 400.8 ± 0.1 eV, and vibrational separation, ω_e = 230 meV, are characteristic of gaseous nitrogen and are in close agreement with literature[3]. Furthermore, the significantly broadened peaks (FWHM) observed are a consequence of the additional decay channels available upon incorporation, from free gas, too deep within the TiN film's interstitials[4].

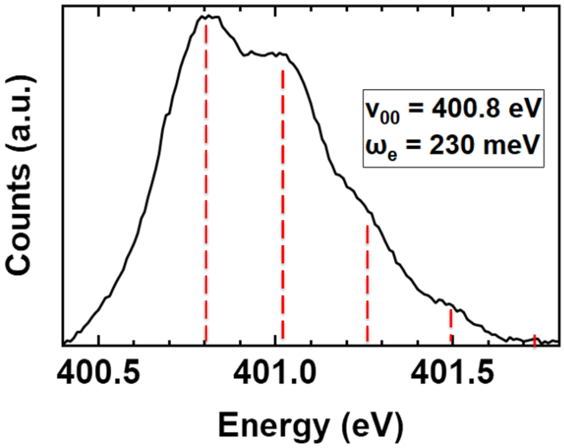

Figure S5. NEXAFS spectrum resolving the vibrational oscillations of interstitially trapped molecular nitrogen (N_2 gas flow = 12.5%, sample E).

Endurance measurement of TiN/HZO/TiN MIM structure

Figure S6 compares the endurance data of sample B and a MIM structure of TiN/HZO/TiN deposited with the same processing conditions, to highlight the superior cycling performance of the MIM structure. We observe good FE properties and stable cycling which is sustained over at least 1.8 million cycles.

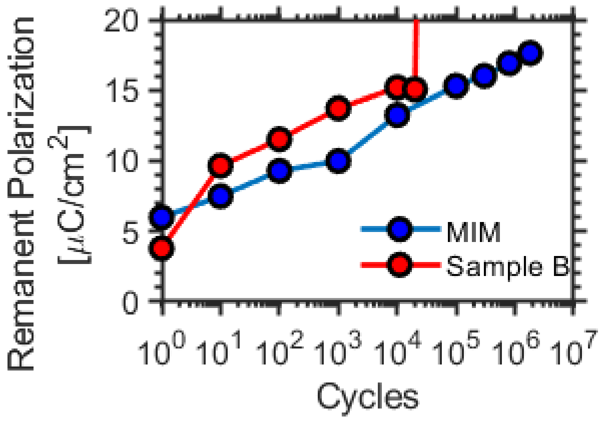

Figure S6. Cycling endurance comparison between sample B and the corresponding MIM structure, deposited by the same processing conditions.

Energy Dispersive X-Ray Spectroscopy

Table S1 summarizes the EDX results of samples C-E measured at an acceleration voltage of 10 kV. The displayed value in **Table S1** is the average of three different locations on the surface of the sample. In supplementary **Figure S7** an EDX spectra of sample C is presented. Since we lack a calibration sample with a pre-determined stoichiometry for the EDX setup the absolute values can be put to question. However, of main importance here are the relative changes in composition between the samples, and these exhibit a distinct trend with increasing nitrogen content of the TiN film with increased N_2 flow during deposition.

Table S1. Deposition Conditions of TE TiN samples.

Sample	Pressure [mTorr]	Ar Flow [sccm]	N_2 Flow [sccm, (%)]	Ti [Atomic %]	N [Atomic %]
C	4.0	14	-	44.9	55.1
D	4.0	12	0.75(6.25%)	42.8	57.2
E	4.0	12	1.5(12.5%)	40.7	59.3

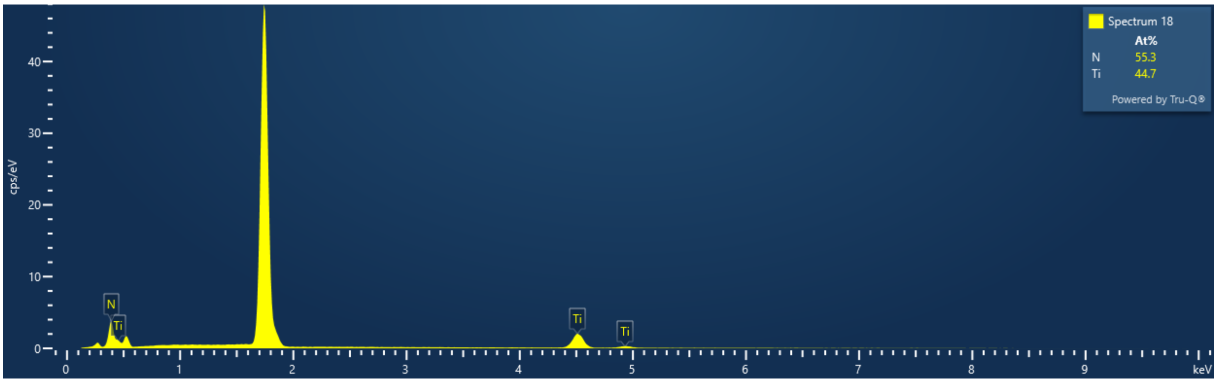

Figure S7: EDX spectra of sample C.

X-Ray Photoelectron Spectroscopy

Table S2 shows relative peak components from all Ti 2p $_{3/2}$ XPS data. The inelastic mean free path was calculated (based on titanium oxide) using the TPP-2M formulism developed by Tanuma et al. [5]. All data was analyzed using IGOR Pro software. For clarity, the Ti 2p $_{1/2}$ split component was cropped, Shirley background was removed, and peaks were coherently fitted with Voigt lineshapes, respecting the observed increasing Gaussian broadening with kinetic energy. Three Ti-related components were observed, with chemical shifts of 0.9 and 1.5 eV between them. In **Figure S8** a typical deconvolution is shown with the constituent components, from sample C at incident beam energy of 1150 eV. We assign the peak at 458.9 eV to TiO_2 with Ti in a 4+ oxidation state, and the peaks at lower binding energy to oxynitride (TiN_xO_y) components. Similar to Filatova et al.[6], we note that oxynitride components may vary in stoichiometry, i.e. in the concentration of oxygen and nitrogen. Furthermore, they might overlap in binding energy with Ti_2O_3. Here, the peaks at 458 and 457.4 eV are generically assigned TiN_xO_y, as their local and oxidized chemical arrangement is of less concern than their qualitative influence on the $Hf_{1-x}Zr_xO_2$ stack. **Figure S9** visualizes the variation of the TiN_xO_y peak components with depth for the three samples, with a higher oxidation level towards the surface due to oxidation from the ambient air. As seen in the absorption data, sample E clearly has more nitride species (TiN_xO_y) and less oxide (TiO_2) indicating that the Ti-N in this sample is more thermodynamically stable and less prone to scavenging oxygen from the environment. Strikingly similar to the NEXAFS spectra in Figure 4, intermediate gas flow (sample D, N_2 flow = 6.25%) deposition exhibits decreased nitride relative to oxide when looking deepest; it is only after N_2 flow is further increased to 12.5% that significantly more nitridation occurs and oxidation is suppressed.

Table S2. Ti 2p $_{3/2}$ Peak Components (%)

Energy (eV)	*458.9*	*458*	*457.4*	*458.9*	*458*	*457.4*	*458.9*	*458*	*457.4*
Assignment	*Ti^{4+}*	*TiN_xO_y*		*Ti^{4+}*	*TiN_xO_y*		*Ti^{4+}*	*TiN_xO_y*	
IMFP (Å)	*Sample C*			*Sample D*			*Sample E*		
6	*67*	*3*	*29*	*67*	*8*	*25*	*62*	*9*	*29*
9	*66*	*4*	*30*	*67*	*11*	*22*	*60*	*12*	*28*
16	*62*	*5*	*33*	*65*	*14*	*21*	*57*	*16*	*27*

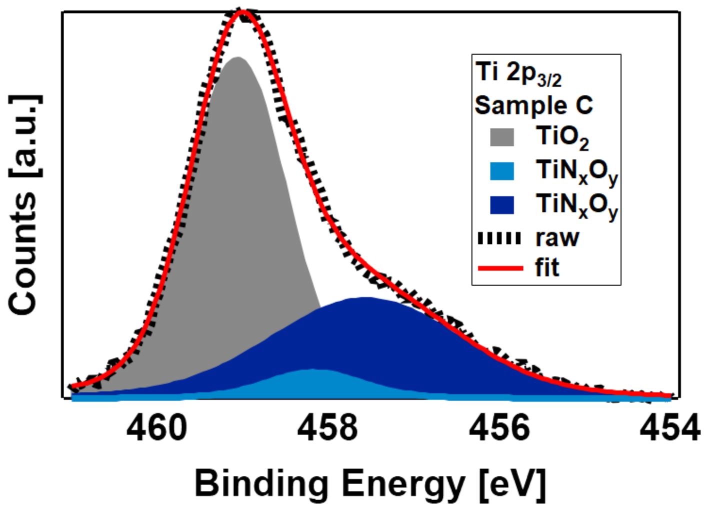

Figure S8. Typical Ti 2p spectral deconvolution used for component comparison (sample C at 1150 eV excitation).

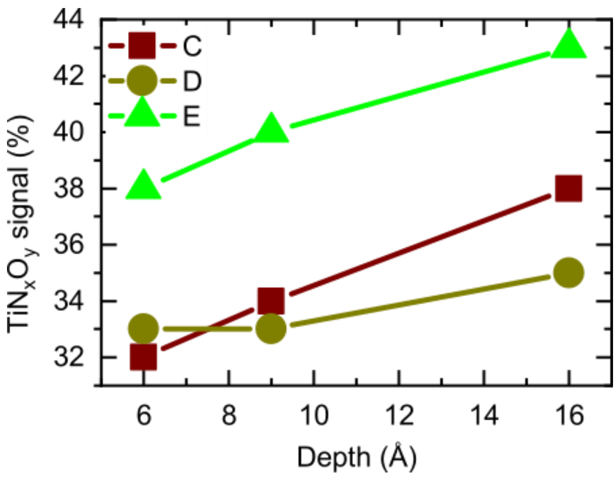

Figure S9. TiN_xO_y XPS signal versus depth (IMFP) of a 2 nm thick TiN film sputtered on $Hf_{1-x}Zr_xO_2$ for samples C-E.

Supporting References:

(1) Petrov, I.; Barna, P. B.; Hultman, L.; Greene, J. E. Microstructural Evolution during Film Growth. *J. Vac. Sci. Technol. A Vacuum, Surfaces, Film.* **2003**, *21* (5), S117–S128.

(2) Schwarzer, N.; Richter, F. On the Determination of Film Stress from Substrate Bending: STONEY's Formula and Its Limits. *Whitepaper* **2006**, No. 1, 1–17.

(3) Chen, C. T.; Ma, Y.; Sette, F. K-Shell Photoabsorption of the N2 Molecule. *Phys. Rev. A* **1989**, *40* (11), 6737–6740.

(4) Petravic, M.; Gao, Q.; Llewellyn, D.; Deenapanray, P. N. K.; Macdonald, D.; Crotti, C. Broadening of Vibrational Levels in X-Ray Absorption Spectroscopy of Molecular Nitrogen in Compound Semiconductors. *Chem. Phys. Lett.* **2006**, *425* (4–6), 262–266.

(5) Tanuma, S.; Powell, C. J.; Penn, D. R. Calculation of Electron Inelastic Mean Free Paths (IMFPs) VII. Reliability of the TPP-2M IMFP Predictive Equation. *Surf. Interface Anal.* **2003**, *35* (3), 268–275.

(6) Filatova, E. O.; Sakhonenkov, S. S.; Konashuk, A. S.; Kasatikov, S. A.; Afanas'Ev, V. V. Inhibition of Oxygen Scavenging by TiN at the TiN/SiO2 Interface by Atomic-Layer-Deposited Al2O3 Protective Interlayer. *J. Phys. Chem. C* **2019**, *123* (36), 22335–22344.
